# END-OF-LIFE PLANNING IN GERMANY AND SOUTH KOREA: PERCEIVED ENGAGEMENT AND ACTUAL BEHAVIOR

**DOI:** 10.1093/geroni/igae098.0370

**Published:** 2024-12-31

**Authors:** Yaeji Kim-Knauss, Yumi Shin, Jung-Hwa Ha, Frieder Lang

**Affiliations:** Friedrich-Alexander-Universität Erlangen-Nürnberg, Nuremberg, Bayern, Germany; Daegu University, Gyeongsan, Republic of Korea; Seoul National University, Seoul, Republic of Korea; Friedrich-Alexander-Universität Erlangen-Nürnberg, Nuremberg, Bayern, Germany

## Abstract

End-of-life planning could manifest differently across cultures, shaped by prevailing social norms. This study examines behaviors associated with perceived engagement in end-of-life planning and explores potential cultural variations. We focus on two distinct cultures, Germany and South Korea, drawing on data from the ‘Ageing as Future’ project’s online survey conducted in 2023 for the German sample (aged 60+, n=708) and data collected between 2023 and 2024 for the Korean sample (aged 60+, n=519). We identify two outcome behaviors of interest: advance care planning (ACP) and discussions with relatives about one’s death, commonly known as formal and informal end-of-life planning, respectively. Logistic regression models revealed that perceiving oneself as actively engaged in end-of-life planning increased the likelihood of completing ACP, a finding consistent across cultures. Additionally, being German, older, female, and having higher education levels were associated with higher odds of ACP completion. Concerning discussions with relatives, on the other hand, perceiving engagement in end-of-life planning increased the likelihood of this informal planning, with a significantly stronger effect observed in the Korean sample compared to the German sample. Being German and having at least one child were also positively associated with engaging in these discussions. These findings suggest a greater reliance on informal discussions about death with relatives in the Korean context. Given the cultural differences and the lower prevalence of end-of-life discussions in Korea compared to Germany, the normalization of such discussions in Korea is needed, but with a culturally tailored approach.

